# Work along Both Lines: The Positive Impact of Work-Based Social Media Use on Job Performance

**DOI:** 10.3390/ijerph182111578

**Published:** 2021-11-04

**Authors:** Junzhe Zhao, Tengfei Guo, Sudong Shang, Minghui Wang

**Affiliations:** 1 Institute of Psychology and Behavior, Henan University, Kaifeng 475004, China; zhaojunzhe97@163.com (J.Z.); guotengfeihnu@163.com (T.G.); 2 Griffith Business School, Griffith University, Southport, QLD 4215, Australia; s.shang@griffith.edu.au

**Keywords:** work-based social media use, work engagement, work interruptions, job performance

## Abstract

Social media has rapidly become an important tool in organizations and has a significant impact on employees’ work and organizational operations. By applying social media to their daily work, employees gain access to important information resources that can help them get things done. Using the conservation of resources theory, this study examines the impact of work resources generated by employees’ work-based social media use on work status, as well as job performance. Data were collected from the employees of Internet companies in Henan Province and Shanghai, China. We distributed 519 pairs of questionnaires, and 369 pairs of valid paired questionnaires were returned. To estimate the proposed relationships in the theoretical framework, we used SPSS and MPLUS. The results show that work-based social media use can increase employees’ work engagement, which in turn increases task performance, job dedication and interpersonal facilitation. It also reduces the negative effects of work interruptions on task performance and job dedication. Therefore, we conclude the positive effects of work-based social media use on job performance can be achieved by increasing work engagement and by reducing work interruptions.

## 1. Introduction

According to the latest statistics released by WeAreSocial and Hootsuite, the number of global Internet users reached 4.66 billion in January 2021, of which about 4.2 billion are social media users [1]. Social media use has pervaded every aspect of people’s lives, and it continues to permeate the workplace, reshaping the way employees work and the way organizations operate. Social media can be very beneficial to an organization, despite the fact that the use of social media dose present some challenge or risks, such as privacy issues, criticisms, distractions [2]. At the organizational level, social media practices facilitate knowledge sharing and accelerate the flow of information and resources within the organization [3]. At the individual employee level, efficient and frequent interactions have facilitated effective communication and collaboration among employees [4]. The popularity and increasing importance of social media has contributed to a greater understanding of the role of technology in organizations. At the same time, this real-world context also provides new challenges for organizational managers and theoretical researchers, namely, how can social media practices be effectively managed? How can the mechanisms in play during social media use be understood?

Social media (SM) refers to a set of Internet applications based on Web 2.0 ideas and technologies that support user content generation and exchange, allowing people to write, share, rate, discuss and communicate with each other. These applications include Facebook, Twitter, WeChat, Weibo, social networking sites and others [5,6]. As research continues, scholars have called out that social media use cannot simply be studied as a unitary construct as this may lead to inconsistent findings [4]. Existing studies classify social media using two main perspectives: purpose and scope. In the first perspective, purpose, the use of social media is divided into work-based social media and social-based social media while the second perspective holds that the scope of use can be divided into internal use and external use [7]. Our paper focuses on social media use during working hours and we examine the work-based social media use of employees and its impact on job performance.

Work-based social media use refers to the use of social media by employees for activities such as information transfer and knowledge sharing to facilitate resource generation, team collaboration, and the exchange of core work information and content [8]. For example, a worker can use social media to organize meetings, schedule appoint-ments, send documents, and communicate about work activities with coworkers in an efficient and timely manner [9]. Previous studies on the mechanisms of social media effects on job performance have mainly involved knowledge sharing and interpersonal relationships [7]. Social media provides an open communication environment that allows employees to share their ideas, opinions, and knowledge freely and easily. This promotes knowledge sharing activities within the organization, which in turn, significantly affects job performance [10,11]. In addition, social media can effectively support employees’ vertical and horizontal communication within the organization, strengthening relationships between superiors and subordinates and the exchanges between team members. These social relationships often translate into dedication to work beliefs and goals, which ultimately improves employee performance [12]. Although existing research has provided some guidance in overall understanding the mechanisms of the effects of social media, there is not formed a clear knowledge framework related to the work-based social media use [13]. Using the conservation of resource theory, our paper seeks to explore how employees’ work-based social media use influences job performance based on two opposing mediating variables: work engagement and work interruptions. This study contributes to a richer understanding of the mechanisms of social media use within a workplace context. In determining how social media use connects with work performance and employee engagement, this paper contributes theoretical and practical knowledge to the field of organizational development.

## 2. Theoretical Foundation and Hypotheses Development

### 2.1. Conservation of Resources Theory

Hobfoll’s [14] theory of resource conversation provides a starting point for understanding the positive effects of social media use. This theory suggests that individuals are driven by the need to maintain or acquire resources, which are things perceived by the individual as being of value to them or which enables them to access what they value. The framework suggests that material resources include cars, properties, and tools needed to carry out work, and their value is derived from their inherent physical properties or the information they contain about an individual’s identity and status. The technological era has enabled the successful integration of social media as an organizational tool into employees’ daily lives, as well as a material resource. The conservation of resource theory enables the positive impact of social media use to be explained in two ways. Firstly, the initial acquisition of resources is beneficial to the progressive acquisition of resources, creating a resource acquisition spiral effect [15]. Social media is a material resource, and the positive consequences of using social media can also be understood as an effective resource, such as accessibility and efficient communication [16]. At the same time, its physical features, such as visibility, editability, persistence, enable employees to learn from the experiences of others and gain more effective resources through vicarious learning [17]. Therefore, employees can access more resources at work through the use of social media [18]. Secondly, employees can use the resources they already have to cope with stressful situations in their current environment or future stressful situations [16]. Therefore, the resources employees have and use by social media use help to mitigate current or future stressful situations (work interruptions). Specifically, on the one hand, employees who have access to adequate resources through social media will use the key resources to deal with the possible problems in the work; on the other hand, employees respond by actively building and protecting their existing reserves of resources to deal with stressful situations, searching for useful information resources on social media in a more proactive way.

### 2.2. Research Hypotheses

Social media is a physical resource that derives its value from its inherent attributes such as its visibility, persistence, and relevance [14]. Employees’ use of social media can generate a new resource embedded in the network, social media capital, to achieve organizational goals [18]. Specifically, social media use facilitates information sharing and the formation of cognition-based instrumental ties [19], which means employees will obtain more resource (e.g., information and knowledge) to accomplish task. When employees have sufficient resources through social media use, it helps to reduce work stress and also makes them more willing to engage in additional behaviors that may fall outside the scope of their job description. Such behaviors may include helping colleagues with their tasks and proactively contributing, which in turn increases interpersonal facilitation and job dedication [20]. Secondly, the introduction of social media in terms of technical features, such as visibility, on the one hand makes it easy for workers to achieve vicarious learning by observing others do and with whom, which can effectively improve their working competence [21]. On the other hand, it can avoid unnecessary work time and repetitive work, thus increasing productivity, which is ultimately reflected in improved task performance [4]. For example, when relevant problems occur, employees can take advantage of and recombine available knowledge to develop the right solutions without undertaking duplicated work [21]. This brings us to our first hypothesis:

**Hypothesis** **1** **(H1).** *Work-based social media use has a positive relationship with employees’ (a) job dedication, (b) interpersonal facilitation, and (c) task performance*.

Based on conservation of resources theory, initial resource acquisition facilitates further resource acquisition. The work resources obtained by employees through the use of social media contribute to further acquisition of psychological resources—work engagement [2]. Specifically, the open environment provided by social media improves exchange and access to information (e.g., information about work, other organizational members and their work tasks), which helps to facilitate efficient communication resources associated with social media use for work among coworkers [16]. As conservation of resources theory predicts, these work resources can potentially enhance employees’ level of engagement [2]. In addition, work communication on social media not only facilitates sharing of work resources, but also plays a critical role in maintain employee engagement by communicating for and about work [22]. Work engagement is a positive, fulfilling state, characterized by energy, dedication, and focus [23]. Employees with high work engagement are willing to invest energy in their work and are able to focus on their work and overcome challenges along the way. In addition, employees will have a stronger sense of engagement at the cognitive and emotional levels and experience more positive emotions such as pride and happiness. In this state of work, employees will have more psychological resources and will be more willing to help others and accept challenges [24]. As such, our second hypothesis is as follows:

**Hypothesis** **2** **(H2).** *Work engagement mediates between social-based social media use and employee (a) job dedication, (b) interpersonal facilitation, and (c) task performance*.

Work interruptions are a common stress in workplaces which can negatively affect the performance of employees [25]. Employees may need to use a great deal of resources to cope with work interruptions—should their resource pool be insufficient, they may develop fatigue and negative emotions [26], The conservation of resource theory holds that those with more resources are less likely to suffer from resource loss [27]. The use of work-based social media by employees can help to reduce the consumption of resources in two ways. First, by leveraging social media for work activities, employees are able to achieve shorter and more efficient interactions [20]. For example, before social media was introduced, managers often send task information using files or letters which caused information delays; but now, managers’ messages can be instantly shared among the employees by social media. This allows work-related issues to be resolved with minimal interruptions, thus avoiding more work interruptions [4]. Second, social media is a visible communication channel, which allows employee to view past interactions and information affords individuals a chance to examine what has been previously successful and to learn from the experiences of others [28]. In other words, employees can gain work and colleague information by observing other’s communication in social media; specifically concerning “who knows what” and “who knows whom” knowledge [17,29]. Visible work-related knowledge empowers employees to maintain necessary connections and reduce waiting time and duplicated work, which helps employees cope with and manage work interruptions caused by irrelevant information [17,30]. Fewer work interruptions give employees sufficient energy to complete their work tasks, contributing to a good working environment, job satisfaction, and greater work engagement [31]. This leads to our third hypothesis:

**Hypothesis** **3** **(H3).** *Work interruptions mediate the relationship between social-based social media use and employees’ (a) job dedication, (b) interpersonal facilitation, and (c) task performance*.

## 3. Research Methodology

### 3.1. Data Collection and Sample

We conducted a survey on middle and junior level employees of Internet companies in Henan Province and Shanghai, China. Questionnaires were distributed in two ways. One was to give participants paper questionnaires to fill out on the spot, and the other was to fill out online through a web link. To avoid possible effects of common method bias and social desirability on the study results, data were obtained from two sources: employees and supervisors. Specifically, employees were required to report only their personal demographic information and to evaluate the work-based social media use, work engagement, and work interruptions. The supervisor only needed to evaluate the employee’s performance. A total of 519 pairs of questionnaires were distributed in this study, and 369 pairs of valid paired questionnaires were returned, with a valid return rate of 71.1%.

Table 1 presents the demographic characteristics of the 369 participants. In terms of the gender ratio of the participants, 41.19% of the participants were male, and 58.81% were female. In terms of age, the majority of the participants were below 30 years old. From the perspective of educational background, 5.96% had graduate degrees or higher, 69.11% had bachelor’s degrees, 23.04% had college degrees, and 1.90% had finished high school or below. From the perspective of the participants’ years of working, most had been working in the company for a period of one to six years.

### 3.2. Measurements

The instruments used in this study were all published and established scales, and a two-way translation process was used for the English version of the scales. All scales were scored on a 7-point Likert scale, with 1 indicating “strongly disagree” and 7 indicating “completely agree”.

#### 3.2.1. Work-Based Social Media Use

We used a five-item work-based social media use scale to capture data on social media use by employees for work-related activities (e.g., “I use social media to set up meetings with colleagues about work projects”) [32]. The Cronbach’s alpha coefficient in this study was 0.81.

#### 3.2.2. Work Engagement

We used a nine-item work engagement scale to measure the work engagement of employees after using social media for work (e.g., “At my work, I feel bursting with energy”) [33]. The Cronbach’s alpha coefficient in this study was 0.91.

#### 3.2.3. Work Interruptions

We used a three-item work interruptions scale to capture work disruptions perceived by employees in organizational contexts (e.g., “Using social media tools inhibits my concentration on work”) [34]. Since the Cronbach’s alpha of the work interruptions is 0.61 which only meets the minimum criteria, we demonstrate the reliability and validity of the scale by combining the recommendations of a with the composite reliability (CR) and average variance extracted (AVE) [35,36]. The CR of work interruptions was 0.80 which was higher than 0.70, and AVE value was 0.57 crossed the threshold of 0.5.

#### 3.2.4. Job Performance

We used a fourteen-item job performance scale to capture three aspects of employees’ task performance, job dedication and interpersonal facilitation at work (e.g., “He/she takes the initiative to solve problems at work”) [37]. Five items on the scale related to task performance and interpersonal facilitation respectively and four items pertained to job dedication. The Cronbach’s alpha coefficients for the three dimensions in this study were 0.84, 0.82, and 0.79, respectively, when employee performance was measured using other ratings. 

We also controlled for certain demographic variables as they can have an impact on employees’ work status and job performance [38,39]. According to previous studies, personal and work resources directly affect work engagement levels and performance [40]. Differences in physical ability and energy can occur depending on the age and gender of the employee. In addition, differences in employees’ gender, age, education, and years of experience can affect their access to resources at work, which in turn affects their work status and performance levels. Given this, we used employees’ gender, age, education, and years of experience at work as control variables [31].

## 4. Results

### 4.1. Common Method Bias

We illustrated common method bias did not have a serious impact on the study in two ways. On the one hand, we programmatically reduced the impact of common methodological biases by collecting data in a supervisor-employee matching fashion. On the other hand, the results of the Harman one-way test showed that the variance explained by the largest factor was 26.07%, which did not reach half of the total explanation (57.95%) and initially indicated that there was no serious problem of common method bias. The results based on the unmeasured single-method latent factor showed that the average variance extraction of homologous variance in the model was 0.22, which was lower than the 0.50 standard for homologous variance to be judged as a latent factor [41].

### 4.2. Confirmatory Factor Analyses

We used MPLUS 7.4 (MPLUS 7.4 is a data analysis and statistical software developed by MUTHEN & MUTHEN located in Los Angeles, America) to perform a confirmatory factor analysis of work-based social media use, work engagement, work interruptions, task performance, job dedication and interpersonal facilitation. Specifically, we use latent variable modeling to calculate the fit of various models, and the results are shown in Table 2. The comparative results indicated that among the factor models, the six-factor model had the best fit and good discriminant validity among the six variables.

### 4.3. Descriptive Statistics and Correlations

The means, standard deviations, and correlation coefficients for each variable are presented in Table 3. Work-based social media use was significantly associated with the mediating and outcome variables, setting the stage for the main and mediating effect tests.

### 4.4. Hypothesis Testing

We used MPLUS to construct structural equation models to test the hypotheses and the results showed that work-based social media use significantly influenced work engagement (β = 0.29, *p* < 0.001), and that work engagement significantly influenced job dedication (β = 0.40, *p* < 0.001), interpersonal facilitation (β = 0.27, *p* < 0.001) and task performance (β = 0.30, *p* < 0.001).The results show that the confidence interval of work engagement in work-based social media use with work dedication was [0.058, 0.180], [0.027, 0.146] for interpersonal facilitation, and [0.037, 0.148] for task performance—none of which contained 0 (see Table 4). Therefore, the indirect effect was significant. This shows that work engagement mediates between work-based social media use and job dedication, interpersonal facilitation, and task performance.

**Figure 1:**
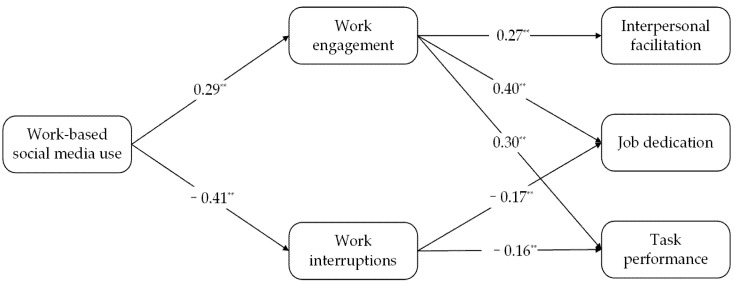
Results of structural equation modeling test. Notes: ** *p* < 0.01.

In addition, the findings also indicated that work interruptions mediated the relationship between job dedication and task performance. Work-based social media use significantly influenced work interruptions (β = −0.41, *p* < 0.001), and work interruptions significantly influenced job dedication (β = −0.17, *p* < 0.05) and task performance (β = −0.16, *p* < 0.05). The results show that work interruptions in work-based social media use have a confidence interval of [0.002, 0.146] with job dedication, and [0.003, 0.140] with task performance, both of which do not contain 0 (see Table 4). The specific results are arranged in Figure 1.

## 5. General Discussion

Social media is now a well-established organizational tool that provides links, navigation, and other instructional information for employees to quickly grasp. By using social media at work, employees can access the various resources needed to complete their tasks [18]. This study proposes a model based on the conservation of resource theory to illustrate the mechanisms by which work-based social media use affects job performance in two ways: increasing positive effects (increased work engagement) on the one hand and decreasing negative effects (decreased work interruptions) on the other.

### 5.1. Theoretical Implications

This study explores the impact of work-based social media use on job performance from a resource-based perspective, further enriching the empirical research on the role of social media influence. Although studies have explored the impressions of social media use and found significant effects of work-based social media use on knowledge sharing, work motivation, and the socialization of new employees [4,42,43], the impact of work-based social media use on job performance remains less well understood. This study proposes and tests the hypothesis that work-based social media use positively affects employee performance based on the conservation of resource theory. This study found that work engagement mediates the relationship between work-based social media use and interpersonal facilitation, job dedication, and task performance. Consistent with previous research, work-based social media use is effective in facilitating two-way communication between employees, and employees are more engaged in their work tasks and roles in a work environment when they can freely share their ideas and perspectives [2]. Employees with high work engagement are more inclined to be passionate about their work and to perform well [24]. Implementing social media use within organizations can increase employee engagement by building a positive work environment and improving communication, and this positive work state enables employees to be open to challenges, and to help others while accomplishing performance goals.

Work-based social media use can reduce resource consumption in organizational contexts and mitigate negative outcomes. The study found that work interruptions mediate the relationship between work-based social media use and job dedication and task performance. Employees’ work-based social media use can effectively reduce interruptions in the workplace, which in turn can facilitate the concentration of employees, leading to improved task performance. We explain this in two ways. First, social media provides a new form of communication that helps employees better manage distractions. Unlike the directness of face-to-face communication, employees can choose when to respond and to prioritize their tasks and responses accordingly [30]. Second, message transparency and network translucency of social media use can help to solve unstructured and ill-defined problems through enhancing communication among coworkers [7], thus minimizing the negative impact of work interruptions on performance. A direct result of reduced work interruptions is a reduction in the resources employees need to consume to cope with interruptions. Employees who have abundant resources are better able to cope with stresses related to work, reduce negative emotions such as anxiety, shape a positive work environment, and motivate employees to engage in more contributing behaviors.

### 5.2. Managerial Implications

This study has significant value and implications for managing social media use and developing the value of social media within organizations. It also offers managers insights into their employees’ social media use. When employees use social media in their daily work activities, this can increase their work engagement and reduce their work interruptions. Subsequently, this can further improve task performance, promote concentration on tasks, and improve interpersonal facilitation, which enhances the overall performance of employees. The proliferation of social media is such that managers would do well to harness its power to promote employee engagement and work performance. Managers could conduct training on the use of social media for work in order to take full advantage of the potential offered by social media. Managers could also explore ways to use social media as a mechanism for ensuring that employees have sufficient resources to cope with their work tasks and to improve their work performance.

### 5.3. Limitations and Future Research

This study has certain limitations and shortcomings. A cross-sectional approach was used in this study, which precludes us from establishing a clear causal relationship. Future studies can use a longitudinal or experimental approach to verify the causal relationship between the variables we have identified and to improve on the internal validity of the study. Another limitation of this study is that it focused on Chinese research subjects. As the intensity of social media use and types of social media uptake varies across countries, there may potentially be some bias in the findings of this study [44]. Future research will need to collect data in different countries to further extend the validity of this study. Thirdly, this study only confirms that work-based social media use has a positive impact on job performance but ignores the potential negative effects and risks [9,45]. In particular, as social media is a relatively open information platform, the issue of privacy leaks may be more common [46]. This phenomenon can have a negative impact on employees and even on the organization. Future research should therefore explore the impact of social media from a more holistic perspective, with a view to providing effective management-level advice to avoid these negative effects. Finally, although we also controlled for demographic variables in our study, we did not explore the effect of these variables on the results in depth. Future studies should take these variables into account for important considerations, such as the effects of gender and age differences on social media use and outcomes [31,47]. 

## 6. Conclusions

Social media use in organizations has become a common phenomenon and has a significant impact on employees’ work attitudes and behaviors. Our research shows that the positive effects of work-based social media use on job performance can be achieved by increasing work engagement and by reducing work distractions. This finding implies that managers need to pay more attention to the important role played by social media use in a workplace setting and to take steps to fully utilize its value.

## Figures and Tables

**Table 1:** Demographic characteristics.

Characteristics		Frequency	Percentage (%)
Gender	Male	152	41.19
	Female	217	58.81
Age	20–25	121	32.79
	26–30	169	45.80
	31–35	48	13.01
	36–40	20	5.42
	41–45	7	1.90
	45 or above	4	1.08
Education	Senior high school or below	7	1.90
	Junior college	85	23.04
	Bachelor’s degree	255	69.11
	Postgraduate or above	22	5.96
Years of working	1 or below	40	10.84
	1–3	111	30.08
	4–6	130	35.23
	7–10	48	13.01
	10 or above	40	10.84

**Table 2:** Results of differentiated validity test and common method bias analysis.

Model	χ^2^	df	χ^2^/df	CFI	TLI	RMSEA	SRMR
One-factor model: I1+ I2+ I3+ D1+ D2+ D3	2000.69	275	7.28	0.55	0.51	0.13	0.12
Two-factor model: I1; I2+ I3+ D1+ D2+ D3	1453.54	274	5.30	0.69	0.66	0.11	0.09
Three-factor model: I1; I2; I3+ D1+ D2+ D3	1291.87	272	4.75	0.73	0.71	0.10	0.08
Four-factor model: I1; I2; I3; D1+ D2+ D3	620.75	269	2.31	0.91	0.90	0.06	0.05
Five-factor model: I1; I2; I3; D1; D2+ D3	542.74	265	2.05	0.93	0.92	0.05	0.05
Six-factor model: I1; I2; I3; D1; D2; D3	334.47	256	1.31	0.98	0.98	0.03	0.04

Note: N = 369; I1, work-based social media use; I2, work interruptions; I3, work engagement; D1, job dedication; D2, interpersonal facilitation; D3, task performance; “+” represents two factors combined into one factor.

**Table 3:** Means, standard deviations, and correlations for all included variables.

Variables	1	2	3	4	5	6	7	8	9	10
1 Gender	—									
2 Age	−0.13 *	—								
3 Education	0.09	−0.20 **	—							
4 Work time	−0.10	0.76 **	−0.24 **	—						
5 Work-based social media use	0.09	0.06	−0.05	0.11 *	—					
6 Work engagement	0.04	0.18 **	−0.06	0.18 **	0.26 **	—				
7 Work interruptions	0.02	−0.11	0.08	−0.10	−0.31 **	−0.26 **	—			
8 Job dedication	0.02	0.07	−0.04	0.07	0.12*	0.37 **	−0.19 **	—		
9 Interpersonal facilitation	0.01	0.13 *	−0.04	0.15 **	0.14 **	0.30 **	−0.18 **	0.62 **	—	
10 Task performance	0.07	0.03	−0.07	0.10	0.12 *	0.32 **	−0.21 **	0.66 **	0.61 **	—
Mean	1.59	3.01	3.74	2.83	5.64	5.70	3.45	5.67	5.91	5.99
SD	0.49	1.01	0.70	1.13	0.99	0.77	1.10	0.77	0.68	0.64

Note: N = 369; * *p* < 0.05, ** *p* < 0.01.

**Table 4:** Results of the multiple mediating effects test.

Model	Estimate	SE	Bootstrap(95% CI)
Indirect effects 1			
work-based social media use→work engagement→job dedication	0.11 **	0.03	(0.058, 0.180)
work-based social media use→work engagement→interpersonal facilitation	0.08 *	0.03	(0.027, 0.146)
work-based social media use→work engagement→task performance	0.09 **	0.03	(0.037, 0.148)
Indirect effects 2			
work-based social media use→work interruptions→job dedication	0.07	0.04	(0.002, 0.146)
work-based social media use→work interruptions→interpersonal facilitation	0.05	0.04	(−0.029, 0.134)
work-based social media use→work interruptions→task performance	0.07	0.04	(0.003, 0.140)
Total indirect effects			
work-based social media use→M→job dedication	0.18 **	0.05	(0.103, 0.279)
work-based social media use→M→interpersonal facilitation	0.13 **	0.04	(0.050, 0.206)
work-based social media use→M→task performance	0.15 **	0.04	(0.078, 0.241)
Direct effects			
work-based social media use→job dedication	−0.09	0.08	(−0.228, 0.083)
work-based social media use→interpersonal facilitation	0.02	0.08	(−0.111, 0.188)
work-based social media use→task performance	−0.05	0.08	(−0.185, 0.126)

Note: * *p* < 0.05, ** *p* < 0.01; M, work engagement and work interruptions.

## Data Availability

Not applicable.
